# We Asked the Experts: The WHO Surgical Safety Checklist and the COVID-19 Pandemic: Recommendations for Content and Implementation Adaptations

**DOI:** 10.1007/s00268-021-06000-y

**Published:** 2021-02-26

**Authors:** Nikhil Panda, James C. Etheridge, Takshveer Singh, Yves Sonnay, George Molina, Barbara K. Burian, Nina Capo-Chichi, Christy E. Cauley, David A. H. de Beer, Miliard Derbew, Roger D. Dias, Mary C. Fearon, Mekdes Daba Feyssa, Kathryn Hagen, Manoj Kumar, Tihitena Negussie Mammo, Edward R. Mariano, Alan Merry, Barbara Mushayandebvu, Mary T. Nabukenya, Milind Shah, Lisa Spruce, Thomas G. Weiser, Mary E. Brindle

**Affiliations:** 1 Ariadne Labs, Harvard T.H. Chan School of Public Health, Brigham and Women’s Hospital, University of Calgary, Boston, MA 403-826-7913 USA; 2 Department of Surgery, Massachusetts General Hospital, Boston, MA USA; 3 Department of Surgery, Brigham and Women’s Hospital, Boston, MA USA; 4 Department of Surgery, Cumming School of Medicine, University of Calgary, Calgary, AB Canada; 5 Human Systems Integration Division, NASA Ames Research Center, Moffett Field, CA USA; 6 Department of Pediatric Surgery, Centre National Hospitalier Universitaire Hubert Koutoukou Manga, Cotonou, Benin; 7 Lifebox Foundation, London, UK; 8 Department of Anesthesia, Great Ormond Street Hospital for Children, London, UK; 9 Department of Surgery, School of Medicine, College of Health Sciences, Addis Ababa University, Addis Ababa, Ethiopia; 10 STRATUS Center for Medical Simulation, Brigham and Women’s Hospital, Boston, MA USA; 11 Association of periOperative Registered Nurses, Denver, CO USA; 12 Department of Obstetrics and Gynecology, Saint Paul Hospital Millennium Medical College, Addis Ababa, Ethiopia; 13 Department of Anesthesia, Auckland City Hospital, Auckland, New Zealand; 14 New Zealand Society of Anesthetists, Wellington, New Zealand; 15 Department of General Surgery, Aberdeen Royal Infirmary, Aberdeen, UK; 16 Scottish Mortality and Morbidity Program, Healthcare Improvement Scotland, Edinburgh, UK; 17 Department of Surgery, Addis Ababa University, Addis Ababa, Ethiopia; 18 Anesthesiology and Perioperative Care Service, Veterans Affairs Palo Alto Health Care System, Palo Alto, CA USA; 19 Department of Anesthesiology, Perioperative and Pain Medicine, Stanford University School of Medicine, Palo Alto, CA USA; 20 Department of Anaesthesiology, School of Medicine, University of Auckland, Auckland, New Zealand; 21 Peter Lougheed Hospital, Calgary, AB Canada; 22 Operating Room Nurses Association of Canada, Bath, ON Canada; 23 Department of Anesthesia, Makerere University College of Health Sciences, Kampala, Uganda; 24 Naval Maternity and Nursing Home, Solapur, India; 25 Federation of Obstetric and Gynecological Societies of India, Mumbai, India; 26 Department of Surgery, Stanford University, Palo Alto, CA USA

## Abstract

**Background:**

As surgical systems are forced to adapt and respond to new challenges, so should the patient safety tools within those systems. We sought to determine how the WHO SSC might best be adapted during the COVID-19 pandemic.

**Methods:**

18 Panelists from five continents and multiple clinical specialties participated in a three-round modified Delphi technique to identify potential recommendations, assess agreement with proposed recommendations and address items not meeting consensus.

**Results:**

From an initial 29 recommendations identified in the first round, 12 were identified for inclusion in the second round. After discussion of recommendations without consensus for inclusion or exclusion, four additional recommendations were added for an eventual 16 recommendations. Nine of these recommendations were related to checklist content, while seven recommendations were related to implementation.

**Conclusions:**

This multinational panel has identified 16 recommendations for sites looking to use the surgical safety checklist during the COVID-19 pandemic. These recommendations provide an example of how the SSC can adapt to meet urgent and emerging needs of surgical systems by targeting important processes and encouraging critical discussions.

## Introduction

The World Health Organization (WHO) Surgical Safety Checklist (SSC) was designed to support surgical teams in improving patient safety across the globe. Over 70% of operating rooms (ORs) in 94 countries utilize the SSC in routine surgical care [1]. The SSC improves surgical outcomes by aiding teams in performing key actions and engaging in critical conversations [2, 3]. While the SSC provides a tool for ensuring safety, the pandemic presents unique challenges to the ways in which surgical teams function. As surgical systems are forced to adapt and respond to new challenges, so should the patient safety tools within those systems.

We sought to determine how the WHO SSC might best be adapted during the COVID-19 pandemic.

## Methods

Our team was assembled from Ariadne Labs and Lifebox collaborators. Ariadne Labs, a health systems innovation center at Brigham and Women’s Hospital and Harvard T.H. Chan School of Public Health, was founded by the developers of the original 19-item WHO SSC and benefits from a worldwide network of surgical safety experts and checklist researchers. Lifebox, a global non-profit that partners with providers in low-resource settings to improve surgical safety, facilitated access to surgical and anesthetic leaders from low- and middle-income countries. Of 57 experts invited, we targeted 15 to 20 as panelists.

We used a modified Delphi technique to build consensus for recommended adaptations to the SSC [4]. This process included three rounds: (1) a qualitative round to identify potential recommendations; (2) a quantitative round to assess agreement with proposed recommendations; and (3) an additional quantitative round to address items not meeting consensus.

In the first round (May 2020), experts were invited to suggest SSC content and implementation adaptations through open-ended questions. Responses were organized into a survey for the second round (July 2020). Panelists indicated their agreement with each recommendation via four-point Likert-style questions (“strongly agree,” “agree,” “disagree,” and “strongly disagree”). A fifth option, “important, but not for inclusion on WHO SSC,” was added for content questions.

Likert-style responses were dichotomized to “include” or “exclude.” Consensus for inclusion or exclusion was defined as 70% agreement or higher. Panelists from round two were provided with a survey of items without consensus for the third round (September 2020).

## Results

Of 57 individuals invited to participate, 18 panelists completed round three. The final panel included eight surgeons, five anesthesiologists, three perioperative nurses, and two human factors engineers from eight countries across five continents. Twelve panelists (67%) were from high-income countries and ten (56%) were female.

Twenty-nine suggestions emerged in the first round (19 content items and 10 implementation items). Twelve items reached consensus for inclusion in round two and an additional four were included after round three (Table 1). Three items reached consensus for exclusion.

**Table 1:** Consensus recommendations for SSC content modification and implementation

Consensus recommendations for modifications to content
Review the patient's COVID-19 test results, symptoms, and/or risk factors
Review the plan for intubation
Review the aerosolization risks of the procedure
Confirm that all members of the operating room are wearing appropriate PPE
Ensure in-room availability of all necessary equipment to minimize the number of times individuals enter or leave the operating room
Discuss handling, packaging, and transport of laboratory or pathology specimens
Confirm appropriate postoperative bed availability
Sign out prior to extubation

Consensus recommendations for implementation
Implementation should be rapid and abbreviated given the emergence of the COVID-19 pandemic
Leadership (institutional and national) should encourage implementation of checklist adaptations
Local leaders from surgery, anesthesia, and nursing should be identified for implementation
Local implementation teams should guide site-specific content changes and implementation efforts
More formal and frequent teamwork and communication training with COVID-19 specific simulation should be required
The checklist should be revised periodically as the pandemic progresses
A process for de-implementation of the adapted checklist should be developed

### Content adaptations

Round one recommendations concerning COVID-19 screening and personal protective equipment (PPE) were nearly universal. Other recommendations centered around OR process modifications to minimize the risk of staff exposure.

In round two, three recommendations related to infectious risk assessment achieved consensus for inclusion: review of the patient’s COVID-19 screening or risk, intubation plan, and aerosolization risks. A recommendation to review equipment availability to limit ingress and egress from the OR also reached consensus for inclusion, while a suggestion to review COVID-19 screening of OR team members was rejected.

Four additional recommendations were included after the third round: ensuring the OR team has proper PPE, confirming postoperative bed availability, discussing proper handling of laboratory and pathological specimens, and signing out prior to extubation. Two recommendations reached consensus for exclusion (restriction of OR team to essential staff and use of trocar sealers). Eight recommendations did not achieve consensus. Free text responses indicated that these recommendations were not universally feasible and were covered by other safety instruments, or should be addressed before the patient enters the OR.

### Implementation adaptations

Eight implementation recommendations achieved consensus for inclusion in the second round. These recommendations were focused on the need for rapid implementation, periodic review, and consideration for de-implementation of a pandemic-oriented SSC. Recommendations related to local ownership and leadership support for the adapted checklist were also included, as it was the need for additional training during implementation. Two items reached consensus for exclusion and two did not achieve consensus (specific recommendations for introducing and de-implementing changes and use of electronic displays). These were felt to be potentially useful but not universally necessary or feasible. A recommendation for a separate supplementary COVID-19 checklist which achieved consensus in round two was removed after further discussion with the panelists.

## Discussion

We identified eight content and seven implementation recommendations for SSC adaptations.

Surgical and anesthetic organizations worldwide have created recommendations for restructuring surgical care delivery during the pandemic. These recommendations have primarily focused on considerations at the level of health systems operations (e.g., timing of elective care) [5]. Other tools, like the COVID-19 Surgical Patient Checklist, help programs address system requirements for teams operating during the pandemic [6].

In contrast, our collaborative identified recommendations for adaptations of the content and implementation of the WHO SSC to address the challenges of the global pandemic.

The success of healthcare innovation relies on collaborative implementation with centralized support and local implementation teams. Our team emphasized these hallmarks of successful implementation, but also emphasized that implementation should occur rapidly and remain fluid to accommodate the evolving pandemic.

Several proposed adaptations for content and implementation to the SSC achieved consensus for exclusion or did not achieve consensus (e.g., limiting trainee exposure). Items that did not reach consensus were redundant with other items (e.g., COVID-19 symptom screening in addition to testing) and did not represent a checklist adaptation (e.g., ventilator filter status); or reflected capabilities unique to individual health systems (e.g., electronic display of checklists).

The WHO SSC was designed to be tailored to contextual needs. This set of recommendations is designed to integrate directly into WHO SSCs currently in use. The impact of these recommendations remains to be studied. However, the recommendations provide an example of how the SSC can adapt to meet urgent and emerging needs of surgical systems by targeting important processes and encouraging critical discussions.
